# Sunlight and Health

**Published:** 1884-09

**Authors:** 


### SUNLIGHT AND HEALTH.

FOR SOME considerable time past, the Astronomer Royal and his assistants have been weekly reporting the significant fact that the recorded sunshine during the seven days has been, upon an average, *nil*. Prima facie it is only photographers who need be affected by this intelligence. What can it possibly matter to the world at large if there is not sunshine enough about, to discolor a piece of sensitivized paper? As a matter of fact, however, the discoloration of sensitivized paper is but one of many processes due to the chemical energy of the sunlight. And a prolonged absence of the sunlight is a very serious matter. Its effects upon the health are direct and perceptible; we get no ozone, and we become dull and listless as if we had been sitting up all night.

When thus out of tone and below par, we are consequently deficient in that vital energy which would otherwise enable us to shake off any ordinary ailment. Nor is this all. Absence of sunlight for any considerable period, is almost always followed by epidemic outbreaks. When the sun is active, filth of all kinds putrefies as it collects. When there is no sunshine the filth collects, accumulates in masses, and ferments. These fermented accumulations are a source of positive denger as soon as the sun resumes its activity.

Decomposition under a bright sun is comparatively harmless. Slow decomposition in the dark is especially hostile to health. We need no chemist to tell us all this; but. at the same time it is as well to bear the chemistry of common life in mind. When the Astronomer Royal reports a total absence of sunshine, we ought to be especially careful; and, it may be added, children suffer more from the loss of the sun's rays than do adults. Adults have only to keep alive; children have to keep alive and to grow, which entails a double amount of chemical work. Now, if there be no sunshine, we can best supplement its absence by exercise. And yet, strangely enough. the absence of sunshine is regarded by most mothers as a sufficient ground for keeping children within doors. It is, on the contrary, the very reason why they should be sent out and kept out as much as possible.

---

Berlin, in 1816, had a population of 195,000; London had one of 958,863, and Paris one of 713666 Sixty years later, Berlin had 1,250,000, London, 4,000,000, and Paris nearly 2.300,000. Berlin increased 6-fold, London 4-fold, and Paris 3-fold.

